# The Neurological Metabolic Phenotype in Prolonged/Chronic Critical Illness: Propensity Score Matched Analysis of Nutrition and Outcomes

**DOI:** 10.3390/nu17142302

**Published:** 2025-07-12

**Authors:** Levan B. Berikashvili, Alexander E. Shestopalov, Petr A. Polyakov, Alexandra V. Yakovleva, Mikhail Ya. Yadgarov, Ivan V. Kuznetsov, Mohammad Tarek S. M. Said, Ivan V. Sergeev, Andrey B. Lisitsyn, Alexey A. Yakovlev, Valery V. Likhvantsev

**Affiliations:** 1 Federal Research and Clinical Center of Intensive Care Medicine and Rehabilitology, Moscow 107031, Russia; lberikashvili@fnkcrr.ru (L.B.B.); ashest@yandex.ru (A.E.S.); p.polyakov@fnkcrr.ru (P.A.P.); avyakovleva@fnkcrr.ru (A.V.Y.); ikuznecov@fnkcrr.ru (I.V.K.); smtshm@fnkcrr.ru (M.T.S.M.S.); dr.1vansergeev@yandex.ru (I.V.S.); ayakovlev@fnkcrr.ru (A.A.Y.); lik0704@gmail.com (V.V.L.); 2 V. M. Gorbatov Federal Research Center for Food Systems, Russian Academy of Sciences, Moscow 109316, Russia; lis@vniimp.ru

**Keywords:** metabolic state, critical illness, metabolism, stroke, traumatic brain injury

## Abstract

**Background**: Brain injuries, including stroke and traumatic brain injury (TBI), pose a major healthcare challenge due to their severe consequences and complex recovery. While ischemic strokes are more common, hemorrhagic strokes have a worse prognosis. TBI often affects young adults and leads to long-term disability. A critical concern in these patients is the frequent development of chronic critical illness, compounded by metabolic disturbances and malnutrition that hinder recovery. **Objective**: This study aimed to compare changes in nutritional status parameters under standard enteral nutrition protocols and clinical outcomes in prolonged/chronic critically ill patients with TBI or stroke versus such a population of patients without TBI or stroke. **Methods**: This matched prospective–retrospective cohort study included intensive care unit (ICU) patients with TBI or stroke from the Federal Research and Clinical Center of Intensive Care Medicine and Rehabilitology and patients without these conditions from the eICU-CRD database. Inclusion criteria comprised age 18–74 years, ICU stay >5 days, and enteral nutrition. Patients with re-hospitalization, diabetes, acute organ failure, or incomplete data were excluded. Laboratory values and clinical outcomes were compared between the two groups. Propensity score matching (PSM) was used to balance baseline characteristics (age, sex, and body mass index). **Results:** After PSM, 29 patients with TBI or stroke and 121 without were included. Univariate analysis showed significant differences in 21 laboratory parameters and three hospitalization outcomes. On day 1, the TBI/stroke group had higher hemoglobin, hematocrit, lymphocytes, total protein, and albumin, but lower blood urea nitrogen (BUN), creatinine, and glucose. By day 20, they had statistically significantly lower calcium, BUN, creatinine, and glucose. This group also showed less change in lymphocytes, calcium, and direct bilirubin. Hospitalization outcomes showed longer mechanical ventilation duration (*p* = 0.030) and fewer cases of acute kidney injury (*p* = 0.0220) in the TBI/stroke group. **Conclusions:** TBI and stroke patients exhibit unique metabolic patterns during prolonged/chronic critical illness, differing significantly from other ICU populations in protein/glucose metabolism and complication rates. These findings underscore the necessity for specialized nutritional strategies in neurocritical care and warrant further investigation into targeted metabolic interventions.

## 1. Introduction

Brain injuries, particularly acute stroke (AS) and traumatic brain injury (TBI), represent significant clinical challenges due to their high prevalence and severe outcomes. Ischemic stroke constitutes the majority (85%) of AS cases [1,2,3], whereas hemorrhagic stroke, though less frequent, is associated with substantially higher mortality rates (30–50%) [4] and poorer functional recovery (10–20%) [4]. TBI poses a considerable medical and socioeconomic burden, given its substantial incidence, debilitating consequences, and high rates of both temporary and permanent disability—especially among young and middle-aged adults, the most economically active population segment [5,6]. Severe TBI carries an average mortality rate of 39%, with 60% of patients experiencing unfavorable outcomes on the Glasgow Outcome Scale [7].

The long-term consequences of brain injury, including persistent disability, low return-to-work rates, and extended rehabilitation periods, contribute to substantial economic and societal costs [5,6]. Furthermore, severe brain damage frequently leads to chronic critical illness (CCI) [8], a condition first described in 1985 to characterize patients requiring prolonged intensive care [9]. CCI affects 7.6% of intensive care unit (ICU) admissions [10], with growing prevalence in recent years [11,12,13,14]. Approximately 5–10% of mechanically ventilated patients progress to CCI, often following sepsis [15,16]. Epidemiological data estimate CCI incidence at 42.0 cases per 100,000 individuals, with risk escalating with age [17].

Malnutrition is common among patients with TBI and AS; it is closely associated with decreased level of consciousness (the lower the level of consciousness, the more severe the malnutrition) and increased mortality [18,19]. Many researchers emphasize the high importance of early diagnosis of malnutrition and the start of nutrition therapy (up to 48 h after admission) in this category of patients [20,21]. Malnutrition may arise from decreased food intake secondary to dysphagia and immobility, compounded by elevated energy demands due to injury-related stress. This drives the body to mobilize fat and protein reserves for energy, further exacerbating hypermetabolism and hypercatabolism. Malnutrition and hypermetabolic–catabolic syndrome are key complications in AS and TBI, significantly impairing treatment efficacy and prolonging recovery [21,22,23,24]. Adequate nutritional support is essential for successful rehabilitation, yet metabolic disturbances probably vary across different brain injury subtypes, affecting both macronutrient and micronutrient metabolism. These potential variations highlight the need for condition-specific enteral nutrition formulations optimized for brain injury patients. In addition, the vast majority of studies concern the acute phase of TBI and AS. Data on the metabolic patterns of CCI patients are meager. A deeper understanding of metabolic dysregulation in this population is crucial to guide the development of targeted nutritional interventions that may improve clinical outcomes.

This study aimed to identify specific characteristics of nutritional status in patients with prolonged or chronic critical illness (PCI/CCI) following TBI or stroke, compared to PCI/CCI patients without TBI or stroke.

## 2. Materials and Methods

### 2.1. Trial Design

We conducted a matched prospective–retrospective cohort study. The prospective cohort included patients from the Federal Research and Clinical Center of Intensive Care Medicine and Rehabilitology (FRCC ICMR); the retrospective cohort included patients from the open access, multicenter eICU Collaborative Research Database (eICU-CRD) [25].

FRCC ICMR patients were enrolled under a registered protocol on ClinicalTrials.gov (NCT06545825; registered date: 9 September 2024). The trial protocol was approved by the institutional ethics committee (approval number: 2/24/6, approval date: 18 June 2024). The study began on 9 September 2024. As this was a pilot observational study, no formal sample size or power calculations were performed. This study was designed and conducted in compliance with the ethical principles outlined in the Declaration of Helsinki [26]. The protocol followed the SPIRIT 2013 guidelines [27], and reporting adhered to the STROBE guidelines [28]; the completed checklist is provided in Supplementary Table S1.

### 2.2. Selection Criteria and Study Population

FRCC ICMR patients were admitted to the ICU between September 2024 and November 2024. The eICU-CRD database contains comprehensive clinical data on 200,859 patients admitted to 335 ICUs in 208 US hospitals, collected between 2014 and 2015 [25]. All patient data from the eICU-CRD database were anonymized. One of the authors was granted access to the eICU-CRD database (certificate numbers: 56653575, 56653561, valid until 21 June 2026).

Inclusion criteria for patients were (1) age from 18 to 74 years; (2) stay in the ICU prior to enrollment more than 5 days (according to the PCI definition [7]); (3) enteral nutrition support; (4) diagnosis of TBI or stroke (for FRCC ICMR patients only); and (5) written informed consent obtained from the patient or surrogate decision-maker, or approval by a medical ethics committee (for FRCC ICMR patients only).

Patients were excluded if they met any of the following criteria: (1) re-hospitalization; (2) no body mass index (BMI) data; (3) diabetes mellites; (4) acute kidney injury on admission; (5) acute liver failure on admission; (6) shock; (7) positive end-expiratory pressure (PEEP) exceeding 12 mbar; (8) stroke on admission (for eICU-CRD patients only); and (9) TBI on admission (for eICU-CRD patients only).

### 2.3. Data Collection and Outcomes

In order to compare patients with (FRCC ICMR patients) and without (eICU-CRD patients) TBI or stroke, the following parameters were collected: demographic characteristics (age, sex), BMI, laboratory parameters and their dynamics from the 1st to the 20th day of observation (white blood cells (WBCs), hemoglobin, hematocrit, red blood cells (RBCs), lymphocyte, potassium, sodium, calcium, total protein, albumin day, transferrin day, aspartate aminotransferase (AST), alanine aminotransferase (ALT), total bilirubin, direct bilirubin, C-reactive protein (CRP), blood urea nitrogen (BUN), creatinine, glucose, total cholesterol, low-density lipoprotein (LDL), high-density lipoprotein (HDL), and cortisol), and hospitalization outcome, namely ICU mortality, requirement for mechanical ventilation (MV), its duration, use of vasoactive agents, nonfatal cardiac arrest, new-onset arrhythmias, acute kidney injury, and sepsis. The following nutritional assessment scales were also calculated: the Prognostic Nutritional Index (PNI) as 10 × serum albumin (g/dL) + 0.005 × absolute lymphocyte count (per mm^3^) and the Controlling Nutritional Status (CONUT) [29].

### 2.4. Statistical Analysis

Data distribution was assessed using the Shapiro–Wilk test. Quantitative data were presented as N, Me [Q1; Q3], where N represents the number of available observations, and Me [Q1; Q3] represents the median with interquartile range. Categorical variables were reported as N, *n* (%), where N is the total number of cases, *n* represents absolute frequency, and% shows column percentage.

Non-normally distributed continuous variables were analyzed using a nonparametric Mann–Whitney U test (Wilcoxon rank-sum test). For categorical data comparisons, either Pearson’s χ^2^ test or Fisher’s exact test was used where applicable. All tests were two-sided, and a *p*-value < 0.05 was considered statistically significant.

The relationship between the two quantitative indicators was analyzed using a simple linear regression and scatter plots.

Given the inherent confounding bias characteristic of non-randomized studies and potential selection bias, we performed propensity score matching (PSM) to balance baseline characteristics between compared groups (with vs. without TBI or stroke). The propensity score was derived using multivariable logistic regression, with nearest-neighbor matching at a 1:*n* ratio and caliper width of 0.2 standard deviations of the logit propensity score [30] (caliper was 0.16). Matching covariates included age, sex, and BMI. Covariate balance was quantitatively evaluated by calculating standardized percentage bias across covariates (%bias, computed in Stata as 100× standardized mean difference [SMD]) and visually assessed using “love” plots. Successful matching was defined as achieving absolute values of %bias ≤10% for all covariates. Additionally, we examined the distributional balance of propensity scores through “balance” plots to verify matching quality.

The complete statistical evaluation was implemented in IBM SPSS Statistics (v27.0; IBM Corp., Armonk, NY, USA). All PSM procedures and balance diagnostics (including “love” plots and “balance” plots) were performed using Stata 18 (StataCorp, 2023, Stata Statistical Software: Release 18. College Station, TX, USA: StataCorp LLC), utilizing specialized “pscore” and “psmatch2” modules.

## 3. Results

### 3.1. Patient Characteristics

**Figure 1:**
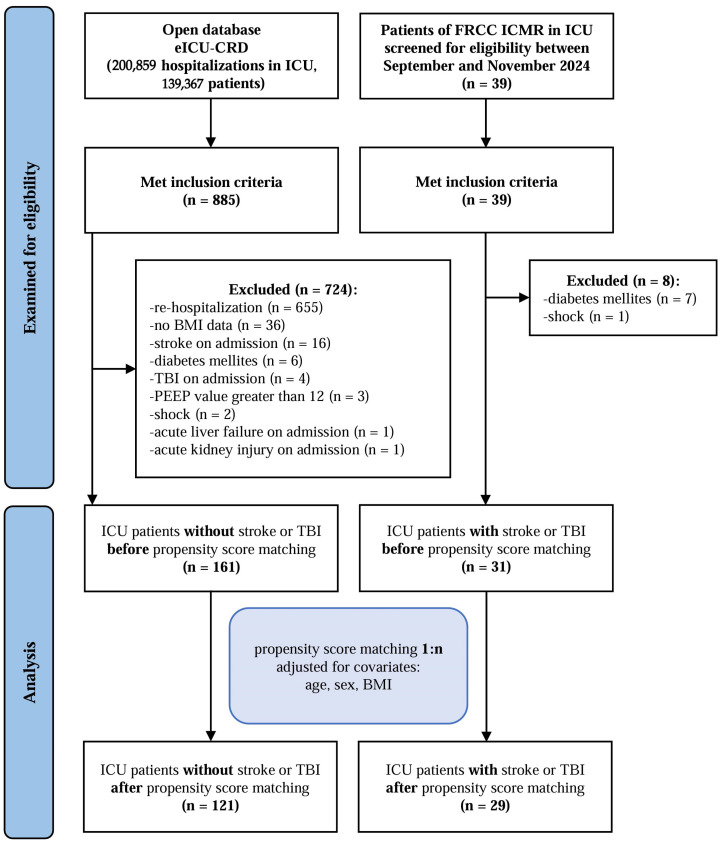
Flowchart of patient selection in the study.

A total of 39 patients were screened for eligibility in FRCC ICMR ICUs between September and November 2024. Following exclusions (*n* = 8), the final study population consisted of 31 patients (21 male; median age 52 years [40–68]). In the eICU-CRD database, 885 patients met the inclusion criteria. After excluding 724 patients, the study cohort comprised 161 patients (89 males; median age 59 years [49–65]). The patient selection process is presented in the flowchart (Figure 1).

Before the PSM procedure, statistically significant differences were observed between patients with and without TBI or stroke in terms of BMI covariate (*p* = 0.016) (Table 1).

**Figure 2:**
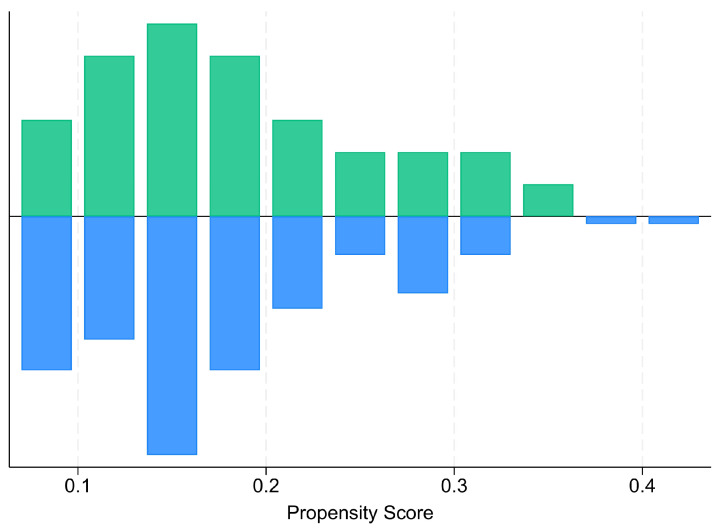
“Balance” plot of propensity score values for eICU-CRD patients without traumatic brain injury or stroke (blue) and FRCC ICMR patients with traumatic brain injury or stroke (green) after propensity score matching.

**Figure 3:**
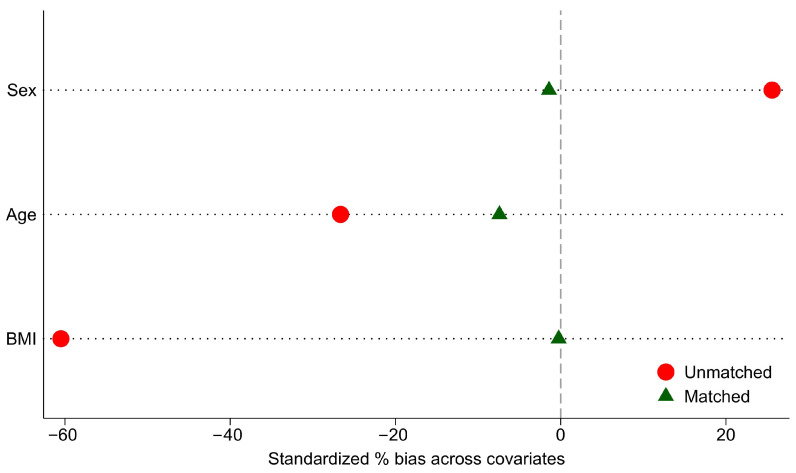
“Love” plot of standardized percentage bias across covariates for unmatched (red balls) and matched (green triangles) groups.

The PSM procedure achieved efficient group balance on all covariates (Figure 2 and Figure 3).

### 3.2. Analyzing the Data After Propensity Score Matching

After PSM, 29 patients remained in the group with TBI or stroke, and 121 patients remained in the group without TBI or stroke.

In the TBI or stroke group, patients with TBI accounted for 27.6% (8/29), those with ischemic stroke for 41.4% (12/29), and those with hemorrhagic stroke for 31.0% (9/29), including two patients with subarachnoid hemorrhage and seven with intracerebral hemorrhage. The median ages in these subgroups were 41 (40; 52), 61 (50; 70), and 55 (47; 69) years, respectively, with no statistically significant differences between them (*p* = 0.921). Univariate analysis revealed statistically significant differences between the two groups in 21 laboratory parameters, the PNI scale, and 3 hospitalization outcomes. At the same time, baseline covariates achieved both statistical and clinical balance (Table 2).

#### 3.2.1. Comparison of Groups on the First Day of Observation

Univariate analysis of first-day observation data after PSM demonstrated that patients with TBI or stroke had significantly higher hemoglobin concentrations (*p* = 0.045), hematocrit level (*p* = 0.016), lymphocyte count (*p* = 0.003), total protein concentrations (*p* = 0.014), and albumin concentrations (*p* = 0.011), as well as significantly lower BUN concentration (*p* = 0.001), creatinine concentrations (*p* = 0.008), and glucose concentration (*p* < 0.001), compared to patients without TBI or stroke. The PNI scale value was statistically significantly higher in the group with TBI or stroke (*p* = 0.006), and the frequency of severe malnutrition (PNI status) was lower (*p* = 0.007; odds ratio [OR] = 4.84, 95% confidence interval [CI]: 1.46–16.06).

#### 3.2.2. Comparison of Groups on 20th Day of Observation

Comparison of groups based on day 20 follow-up data revealed that patients with TBI or stroke had significantly lower calcium concentration (*p* = 0.018), BUN concentration (*p* < 0.001), creatinine concentration (*p* = 0.041), and glucose concentration (*p* = 0.001).

#### 3.2.3. Comparison of Parameter Dynamics

Analysis of 20-day dynamics in laboratory parameters revealed that patients with TBI or stroke exhibited a significantly less pronounced change in lymphocyte count, both in absolute (*p* = 0.009) and relative terms (*p* = 0.008), compared to patients without these conditions. The increase in calcium concentration was also significantly lower in the TBI or stroke group, both in absolute (*p* = 0.030) and relative values (*p* = 0.032). While the total protein concentration decreased from day 1 to day 20 in patients with TBI or stroke, it increased in the comparison group, with statistically significant differences observed in both absolute (*p* = 0.006) and relative changes (*p* = 0.005). The decrease in direct bilirubin was significantly less pronounced in the TBI or stroke group in relative terms (*p* = 0.001). Additionally, the groups differed significantly in glucose dynamics: patients with TBI or stroke had a smaller decrease in glucose concentration, both in absolute (*p* = 0.024) and relative values (*p* = 0.039). The PNI dynamics also showed statistically significant differences between the groups (*p* = 0.047), demonstrating a decrease of 1.3 points in the TBI or stroke group versus an increase of 3.5 points in the non-TBI or stroke group.

#### 3.2.4. Comparison of Hospitalization Outcomes

When analyzing hospitalization outcomes, it was found that patients with TBI or stroke had a statistically significantly longer MV duration (*p* = 0.030), and they less frequently developed acute kidney injury (*p* = 0.022, OR was 4.39 [95% CI: 1.00; 19.28]) during the 20-day observation period of the study. However, simple linear regression analysis demonstrated that the duration of mechanical ventilation did not significantly affect the dynamics of total protein levels from day 1 to day 20 across the entire patient cohort (Table S2 and Figure S1).

## 4. Discussion

### 4.1. Key Findings

This study revealed significant differences in metabolic trajectories and clinical outcomes between critically ill patients with TBI/stroke and those without neurological injury. Initially, the TBI/stroke cohort presented with higher hemoglobin, hematocrit, lymphocyte counts, and protein markers alongside lower renal function parameters and glucose levels. This distinct metabolic profile evolved differently during the 20-day observation period, with the neurological group demonstrating blunted dynamics in lymphocyte responses, calcium regulation, and protein metabolism compared to controls. Particularly noteworthy was the divergent trajectory in protein metabolism: while total protein levels decreased in TBI/stroke patients, they increased in the comparison group. A similar trend was observed for PNI. Although the group of patients without TBI or stroke had a significantly higher proportion of severe malnutrition cases at baseline (90.2% vs. 65.5%), no statistically significant difference remained by day 20 (81.3% vs. 74.1%). This outcome reflects distinct intergroup differences in PNI dynamics: the TBI/stroke group showed a 1.3-point decrease in PNI scores, while the non-TBI/stroke group demonstrated a 3.5-point increase.

The clinical outcomes analysis uncovered important differences in complication patterns. Patients with neurological injuries required significantly longer mechanical ventilation, yet showed substantially lower rates of acute kidney injury (4.39-fold difference). These findings suggest that while TBI/stroke patients face prolonged respiratory support needs, they may be relatively protected against certain systemic complications common in general critical care populations. The observed metabolic patterns, particularly the attenuated fluctuations in lymphocyte counts, calcium, and glucose regulation, may reflect unique adaptive mechanisms in brain-injured patients that warrant further investigation.

### 4.2. Relationship with Previous Studies

Our research represents the first direct comparison of metabolic trajectory differences between PCI/CCI patients with TBI/stroke versus those without neurological injury, using propensity score matching methodology. This approach allowed for rigorous control of confounding variables while examining longitudinal metabolic changes.

We demonstrated a clinically significant divergence in protein metabolism, with TBI/stroke patients showing decreasing total protein concentrations during observation, while non-neurological PCI/CCI patients exhibited increasing levels. This finding aligns with established research documenting hypercatabolic states in brain-injured patients [21,31]. Additionally, serum albumin levels are recognized as an important biomarker of stroke treatment outcomes [32,33]. The progressive protein loss we observed suggests that current nutritional protocols may be insufficient to meet the catabolic demands of neurocritical PCI/CCI patients. These results emphasize the need for more aggressive protein supplementation strategies specifically tailored for this population. The temporal pattern of protein loss provides valuable guidance for timing nutritional interventions.

Moreover, our data reveal attenuated glucose reduction in TBI/stroke patients compared to their non-neurological counterparts, supporting existing evidence of activated glycolysis in brain injury [34,35,36]. This metabolic pattern likely reflects both increased cerebral glucose demands and systemic metabolic reprogramming following neurological injury [34,35]. The blunted glucose fluctuations may represent a protective adaptation to maintain cerebral metabolic homeostasis during prolonged critical illness. These findings challenge conventional glycemic control approaches in neurocritical care, suggesting they may require modification for PCI/CCI patients. At the same time, high triglyceride glucose–body mass index (TyG-BMI) and blood glucose levels significantly affect stroke outcomes [37,38]. In this study, we did not consider patients with diabetes, and at the time of inclusion, patients did not have elevated glucose levels. Patients with impaired glucose tolerance may have other results.

The observed lymphocyte count reduction in TBI/stroke patients confirms and extends previous reports of brain injury-associated immunosuppression in acute care settings [39,40]. Immunosuppression is currently considered an adaptive response that protects the brain from excessive inflammation [41,42]. The pathophysiological mechanisms of this are being actively studied [42,43]. Our study demonstrates that this immunosuppressive effect persists throughout the prolonged/chronic critical illness phase. These findings have important implications for infection risk stratification and immunomodulatory therapies in this vulnerable population. The parallel metabolic and immunological changes we documented may reflect interconnected pathophysiology, which warrants further investigation.

### 4.3. Significance of the Study Findings

The present study provides novel insights into the distinct metabolic and clinical characteristics of prolonged critical illness in patients with TBI or stroke compared to non-neurological critically ill patients. Our findings demonstrate that TBI/stroke patients exhibit a unique metabolic profile, marked by differential protein, glucose, and electrolyte dynamics that persist throughout the clinical course. These metabolic differences likely reflect the specific neuroendocrine and inflammatory responses to brain injury, which appear to modulate systemic complications differently than in general critical care populations.

From a practical standpoint, the documented metabolic trajectories provide critical data for optimizing nutritional support strategies in neurocritical care. The observed protein catabolism patterns, attenuated lymphocyte responses, and distinct glucose metabolism dynamics all point to potentially modified nutritional requirements in brain-injured patients. These findings challenge the assumption that standard critical care nutrition protocols are equally appropriate for neurological and non-neurological ICU populations.

Consequently, this study makes three key contributions to the field: (1) it establishes objective metabolic differences that distinguish TBI/stroke patients in prolonged/chronic critical illness; (2) it identifies their differential risk profile for common ICU complications; and (3) it provides an evidence base for developing targeted nutritional interventions in neurocritical care.

### 4.4. Strengths and Limitations

A strength of this study is the use of a large dataset from the eICU-CRD database, which includes a diverse, multicenter cohort of patients from various intensive care units. Such a multicenter approach enhances the external validity of our findings. Furthermore, the application of propensity score matching effectively minimized the impact of confounders and covariates, allowing for a more balanced comparison between the study groups.

However, several limitations must be acknowledged. The PSM procedure inevitably leads to the loss of some data, potentially reducing the statistical power of the study. Although PSM was used to control for numerous confounders, residual confounders that were not accounted for in the collected data cannot be entirely excluded.

We also cannot state with certainty that the identified characteristics of nutritional status in patients with TBI or stroke are attributable to disease etiology, as they may also be influenced by the observed differences in the duration of mechanical ventilation. Furthermore, the study did not include specific molecular biomarkers associated with stroke or TBI.

### 4.5. Future Studies and Prospects

Further research should validate these metabolic patterns in larger cohorts and investigate targeted nutritional strategies for brain-injured, critically ill patients. Prospective trials are needed to assess whether personalized nutrition protocols can improve clinical outcomes in this population. Additionally, studies should explore the underlying mechanisms of the observed metabolic differences and their relationship to organ protection.

## 5. Conclusions

This study demonstrates that prolonged/chronic critical illness in TBI and stroke patients follows distinct metabolic and clinical trajectories compared to non-neurological critically ill patients. The identified patterns of protein metabolism, glucose regulation, and complication profiles highlight the need for specialized nutritional approaches in neurocritical care. These findings provide a foundation for developing tailored management strategies that may improve outcomes for this vulnerable patient population. Further research should focus on translating these metabolic insights into targeted therapeutic interventions.

## Figures and Tables

**Table 1:** Comparison of patients with and without TBI or stroke before PSM.

Parameters	eICU-CRD Patients Without TBI or Stroke (N = 161)	FRCC ICMR Patients With TBI or Stroke (N = 31)	*p*-Value
† Sex (male), *n* (%)	N = 161; 89 (55.3%)	N = 31; 21 (67.7%)	0.199
† Age, years	N = 161; Me = 59.0 [49.0; 65.0]	N = 31; Me = 52.0 [40.0; 68.0]	0.233
† BMI, kg/m^2^	N = 161; Me = 27.8 [24.2; 33.2]	N = 31; Me = 26.0 [21.6; 29.4]	0.016 *
Laboratory Tests			
WBCs day 1, 10^9^/L	N = 152; Me = 11.2 [7.8; 16.0]	N = 31; Me = 9.9 [6.9; 12.0]	0.036 *
WBCs day 20, 10^9^/L	N = 95; Me = 9.3 [6.3; 11.9]	N = 29; Me = 7.7 [6.3; 9.4]	0.157
Δ WBC day 1–20, 10^9^/L	N = 94; Me = −1.9 [−5.3; 0.6]	N = 29; Me = −1.4 [−2.8; 0.3]	0.233
Δ WBCs day 1–20, %	N = 94; Me = −18.9 [−38.1; 4.9]	N = 29; Me = −16.6 [−29.7; 4.2]	0.368
Hemoglobin day 1, g/L	N = 152; Me = 88.0 [80.0; 100.0]	N = 31; Me = 98.0 [88.0; 114.0]	0.009 *
Hemoglobin day 20, g/L	N = 95; Me = 87.0 [81.0; 97.0]	N = 29; Me = 93.0 [81.0; 103.0]	0.254
Δ Hemoglobin day 1–20, g/L	N = 94; Me = −2.0 [−11.0; 8.0]	N = 29; Me = −5.0 [−18.0; 10.0]	0.192
Δ Hemoglobin day 1–20, %	N = 94; Me = −2.4 [−10.9; 9.2]	N = 29; Me = −6.5 [−17.3; 9.6]	0.192
Hematocrit day 1, %	N = 152; Me = 27.0 [24.8; 31.3]	N = 31; Me = 30.2 [27.6; 33.4]	0.004 *
Hematocrit day 20, %	N = 95; Me = 27.2 [24.8; 30.6]	N = 29; Me = 28.1 [25.6; 32.0]	0.209
Δ Hematocrit day 1–20, %	N = 94; Me = −0.3 [−3.3; 2.3]	N = 29; Me = −2.8 [−5.1; 1.6]	0.120
Δ Hematocrit day 1–20, %	N = 94; Me = −1.0 [−11.5; 9.6]	N = 29; Me = −8.7 [−16.3; 6.0]	0.105
RBCs day 1, 10^12^/L	N = 152; Me = 3.1 [2.7; 3.6]	N = 31; Me = 3.3 [3.0; 3.9]	0.033 *
RBCs day 20, 10^12^/L	N = 95; Me = 3.0 [2.7; 3.3]	N = 29; Me = 3.3 [2.8; 3.6]	0.087
Δ RBCs day 1–20, 10^12^/L	N = 94; Me = −0.1 [−0.4; 0.3]	N = 29; Me = −0.1 [−0.6; 0.4]	0.514
Δ RBCs day 1–20, %	N = 94; Me = −2.3 [−12.8; 10.1]	N = 29; Me = −3.7 [−15.3; 10.1]	0.592
Lymphocytes day 1, 10^9^/L	N = 99; Me = 1.2 [0.7; 1.7]	N = 31; Me = 1.6 [1.2; 2.0]	0.009 *
Lymphocytes day 20, 10^9^/L	N = 61; Me = 1.5 [1.0; 2.2]	N = 29; Me = 1.4 [1.2; 2.0]	0.928
Δ Lymphocytes day 1–20, 10^9^/L	N = 53; Me = 0.2 [0.0; 0.8]	N = 29; Me = 0.0 [−0.5; 0.1]	0.008 *
Δ Lymphocytes day 1–20, %	N = 53; Me = 26.0 [−0.4; 71.3]	N = 29; Me = −1.4 [−27.7; 11.7]	0.008 *
Potassium day 1, mmol/L	N = 152; Me = 3.9 [3.7; 4.2]	N = 31; Me = 3.8 [3.6; 4.0]	0.104
Potassium day 20, mmol/L	N = 96; Me = 4.0 [3.7; 4.4]	N = 29; Me = 3.8 [3.5; 4.1]	0.032 *
Δ Potassium day 1–20, mmol/L	N = 95; Me = 0.1 [−0.4; 0.5]	N = 29; Me = 0.0 [−0.2; 0.2]	0.572
Δ Potassium day 1–20, %	N = 95; Me = 2.5 [−8.2; 13.5]	N = 29; Me = 0.0 [−5.7; 5.3]	0.492
Sodium day 1, mmol/L	N = 152; Me = 140.0 [136.0; 144.0]	N = 31; Me = 138.8 [135.4; 141.7]	0.331
Sodium day 20, mmol/L	N = 96; Me = 138.0 [135.0; 141.0]	N = 29; Me = 137.6 [134.3; 139.5]	0.669
Δ Sodium day 1–20, mmol/L	N = 95; Me = −1.0 [−7.0; 1.0]	N = 29; Me = −0.1 [−6.6; 2.2]	0.469
Δ Sodium day 1–20, %	N = 95; Me = −0.7 [−5.0; 0.8]	N = 29; Me = −0.1 [−4.7; 1.6]	0.452
Calcium day 1, mmol/L	N = 147; Me = 2.1 [2.0; 2.2]	N = 31; Me = 2.0 [2.0; 2.1]	0.441
Calcium day 20, mmol/L	N = 91; Me = 2.2 [2.0; 2.4]	N = 29; Me = 2.1 [1.9; 2.2]	0.009 *
Δ Calcium day 1–20, mmol/L	N = 89; Me = 0.1 [0.0; 0.2]	N = 29; Me = 0.0 [−0.1; 0.1]	0.005 *
Δ Calcium day 1–20, %	N = 89; Me = 5.4 [−1.2; 11.3]	N = 29; Me = 1.3 [−5.1; 4.3]	0.006 *
Total protein day 1, g/L	N = 74; Me = 56.0 [49.0; 61.0]	N = 31; Me = 60.0 [55.7; 66.7]	0.009 *
Total protein day 20, g/L	N = 37; Me = 60.0 [55.0; 67.0]	N = 29; Me = 56.3 [49.5; 60.1]	0.047 *
Δ Total protein day 1–20, g/L	N = 28; Me = 6.0 [−2.0; 11.0]	N = 29; Me = −4.2 [−10.2; 0.7]	0.001 *
Δ Total protein day 1–20, %	N = 28; Me = 11.5 [−3.0; 20.1]	N = 29; Me = −5.9 [−18.0; 1.0]	0.001 *
Albumin day 1, g/L	N = 86; Me = 24.0 [20.0; 28.0]	N = 31; Me = 28.4 [24.2; 33.4]	0.002 *
Albumin day 20, g/L	N = 53; Me = 25.0 [20.0; 31.0]	N = 29; Me = 26.4 [23.3; 30.0]	0.240
Δ Albumin day 1–20, g/L	N = 42; Me = 2.0 [−3.0; 5.0]	N = 29; Me = −0.3 [−3.0; 1.5]	0.139
Δ Albumin day 1–20, %	N = 42; Me = 9.5 [−15.8; 23.1]	N = 29; Me = −0.7 [−10.0; 5.3]	0.160
Transferrin day 1, mg/dL	N = 3; Me = 146.0 [132.0; 181.0]	N = 31; Me = 128.0 [92.0; 157.0]	0.348
Transferrin day 20, mg/dL	N = 2; 152.0. 124.0	N = 29; Me = 119.0 [83.0; 154.0]	ID
Δ Transferrin day 1–20, mg/dL	N = 1; −57.0	N = 29; Me = −1.0 [−31.0; 12.0]	ID
Δ Transferrin day 1–20, %	N = 1; −31.5	N = 29; Me = −0.8 [−23.9; 15.7]	ID
AST day 1, U/L	N = 75; Me = 34.0 [19.0; 68.0]	N = 31; Me = 29.8 [20.4; 40.2]	0.422
AST day 20, U/L	N = 39; Me = 29.0 [18.0; 53.0]	N = 29; Me = 23.6 [20.2; 35.6]	0.710
Δ AST day 1–20, U/L	N = 31; Me = −6.0 [−19.0; 15.0]	N = 29; Me = −0.5 [−12.2; 7.0]	0.673
Δ AST day 1–20, %	N = 31; Me = −17.1 [−40.0; 46.7]	N = 29; Me = −1.8 [−34.4; 74.5]	0.750
ALT day 1, U/L	N = 73; Me = 34.0 [22.0; 75.0]	N = 31; Me = 25.9 [21.4; 39.2]	0.215
ALT day 20, U/L	N = 38; Me = 29.5 [20.0; 56.0]	N = 29; Me = 25.1 [20.7; 39.3]	0.451
Δ ALT day 1–20, U/L	N = 28; Me = 0.0 [−54.0; 10.0]	N = 29; Me = 1.5 [−6.2; 6.5]	0.492
Δ ALT day 1–20, %	N = 28; Me = 0.0 [−58.2; 47.1]	N = 29; Me = 6.4 [−28.9; 41.4]	0.492
Total bilirubin day 1, μmol/L	N = 75; Me = 13.7 [6.8; 27.4]	N = 31; Me = 11.2 [8.0; 16.2]	0.391
Total bilirubin day 20, μmol/L	N = 39; Me = 8.6 [6.8; 15.4]	N = 29; Me = 11.3 [8.1; 13.4]	0.288
Δ Total bilirubin day 1–20, μmol/L	N = 31; Me = −1.7 [−15.4; 0.0]	N = 29; Me = −1.5 [−4.1; 2.5]	0.228
Δ Total bilirubin day 1–20, %	N = 31; Me = −28.6 [−66.7; 0.0]	N = 29; Me = −14.0 [−31.3; 22.6]	0.206
Direct bilirubin day 1, μmol/L	N = 37; Me = 3.4 [0.0; 12.0]	N = 30; Me = 2.7 [1.5; 3.3]	0.390
Direct bilirubin day 20, μmol/L	N = 17; Me = 1.7 [0.0; 5.1]	N = 29; Me = 2.4 [1.3; 3.2]	0.973
Δ Direct bilirubin day 1–20, μmol/L	N = 9; Me = −1.7 [−20.5; 0.0]	N = 28; Me = −0.1 [−1.7; 0.8]	0.044 *
Δ Direct bilirubin day 1–20, %	N = 6; Me = −85.4 [−100.0; −50.0]	N = 28; Me = −1.9 [−52.4; 41.0]	0.001 *
CRP day 1, mg/L	N = 2; 151.0. 180.0	N = 31; Me = 65.2 [28.2; 126.9]	ID
CRP day 20, mg/L	N = 2; 174.0. 1.2	N = 29; Me = 43.1 [30.3; 63.7]	ID
Δ CRP day 1–20, mg/L	N = 0	N = 29; Me = −21.2 [−59.4; 18.6]	n/a
Δ CRP day 1–20, %	N = 0	N = 29; Me = −39.1 [−72.4; 77.3]	n/a
BUN day 1, mmol/L	N = 152; Me = 10.5 [5.7; 17.9]	N = 31; Me = 4.9 [3.2; 10.4]	<0.001 *
BUN day 20, mmol/L	N = 96; Me = 9.3 [5.0; 15.2]	N = 29; Me = 4.5 [2.4; 7.9]	<0.001 *
Δ BUN day 1–20, mmol/L	N = 95; Me = −0.4 [−7.9; 3.2]	N = 29; Me = −0.9 [−5.4; 1.3]	0.899
Δ BUN day 1–20, %	N = 95; Me = −2.9 [−49.2; 46.7]	N = 29; Me = −19.0 [−58.7; 40.6]	0.603
Creatinine day 1, μmol/L	N = 150; Me = 97.2 [53.0; 236.0]	N = 31; Me = 59.1 [49.5; 90.0]	0.005*
Creatinine day 20, μmol/L	N = 94; Me = 88.4 [55.7; 185.6]	N = 29; Me = 59.1 [47.9; 91.2]	0.020*
Δ Creatinine day 1–20, μmol/L	N = 92; Me = −9.7 [−54.4; 9.7]	N = 29; Me = −5.3 [−14.3; 13.2]	0.172
Δ Creatinine day 1–20, %	N = 92; Me = −12.0 [−39.5; 17.3]	N = 29; Me = −8.0 [−18.8; 26.9]	0.222
Glucose day 1, mmol/L	N = 152; Me = 7.2 [5.5; 8.9]	N = 30; Me = 5.2 [4.5; 5.7]	<0.001 *
Glucose day 20, mmol/L	N = 96; Me = 5.9 [5.2; 7.3]	N = 29; Me = 4.8 [4.6; 5.2]	<0.001 *
Δ Glucose day 1–20, mmol/L	N = 95; Me = −0.6 [−1.9; 0.1]	N = 28; Me = −0.2 [−1.1; 0.5]	0.069
Δ Glucose day 1–20, %	N = 95; Me = −8.2 [−25.2; 2.0]	N = 28; Me = −4.3 [−19.0; 14.2]	0.106
Total cholesterol day 1, mmol/L	N = 1; 3.4	N = 30; Me = 3.9 [3.2; 4.5]	ID
Total cholesterol day 20, mmol/L	N = 1; 3.2	N = 29; Me = 3.5 [2.8; 4.8]	ID
Δ Total cholesterol day 1–20, mmol/L	N = 0	N = 28; Me = −0.2 [−1.1; 0.7]	n/a
Δ Total cholesterol day 1–20, %	N = 0	N = 28; Me = −7.4 [−26.6; 18.1]	n/a
LDL day 1, mmol/L	N = 1; 1.8	N = 31; Me = 2.6 [2.1; 3.2]	ID
LDL day 20, mmol/L	N = 1; 1.7	N = 29; Me = 2.4 [1.7; 3.2]	ID
Δ LDL day 1–20, mmol/L	N = 0	N = 29; Me = −0.5 [−0.8; 0.4]	n/a
Δ LDL day 1–20, %	N = 0	N = 29; Me = −17.3 [−34.2; 12.2]	n/a
HDL day 1, mmol/L	N = 1; 1.2	N = 31; Me = 0.7 [0.6; 0.8]	ID
HDL day 20, mmol/L	N = 1; 0.6	N = 29; Me = 0.8 [0.5; 1.0]	ID
Δ HDL day 1–20, mmol/L	N = 0	N = 29; Me = 0.1 [−0.1; 0.2]	n/a
Δ HDL day 1–20, %	N = 0	N = 29; Me = 6.9 [−14.3; 25.4]	n/a
Cortisol day 1, nmol/L	N = 4; Me = 460.8 [282.8; 768.4]	N = 31; Me = 409.7 [319.4; 557.7]	0.783
Cortisol day 20, nmol/L	N = 0	N = 29; Me = 399.9 [255.2; 542.1]	n/a
Δ Cortisol day 1–20, nmol/L	N = 0	N = 29; Me = −54.1 [−153.9; 96.8]	n/a
Δ Cortisol day 1–20, %	N = 0	N = 29; Me = −13.9 [−33.3; 23.0]	n/a
Prognostic Nutritional Index (PNI)			
day 1	N = 65; Me = 30.8 [26.7; 36.9]	N = 31; Me = 37.8 [30.6; 42.0]	0.002 *
day 20	N = 41; Me = 31.9 [27.1; 38.9]	N = 29; Me = 36.0 [28.4; 40.3]	0.355
Δ day 1–20	N = 29; Me = 3.2 [−3.3; 6.8]	N = 29; Me = −0.9 [−3.2; 2.1]	0.101
day 1 status–normal (≥45), *n* (%)	N = 65; 2 (3.1%)	N = 31; 5 (16.1%)	0.034 *
day 1 status–mild malnutrition (40–44.9), *n* (%)	N = 65; 5 (7.7%)	N = 31; 6 (19.4%)	0.167
day 1 status–severe malnutrition (<40), *n* (%)	N = 65; 58 (89.2%)	N = 31; 20 (64.5%)	0.004 *
day 20 status–normal (≥45), *n* (%)	N = 41; 5 (12.2%)	N = 29; 2 (6.9%)	0.691
day 20 status–mild malnutrition (40–44.9), *n* (%)	N = 41; 5 (12.2%)	N = 29; 6 (20.7%)	0.506
day 20 status–severe malnutrition (<40), *n* (%)	N = 41; 31 (75.6%)	N = 29; 21 (72.4%)	0.763
Controlling Nutritional Status (COUNT)			
day 1, score	N = 0	N = 30; Me = 6.0 [4.0; 8.0]	n/a
day 20, score	N = 0	N = 29; Me = 7.0 [5.0; 9.0]	n/a
Δ day 1–20, score	N = 0	N = 28; Me = 0.5 [−1.0; 2.0]	n/a
day 1 status–normal (0–1 score), *n* (%)	N = 0	N = 30; 2 (6.7%)	n/a
day 1 status–mild undernutrition (2–4 score), *n* (%)	N = 0	N = 30; 8 (26.7%)	n/a
day 1 status–moderate undernutrition (5–8 score), *n* (%)	N = 0	N = 30; 14 (46.7%)	n/a
day 1 status–severe undernutrition (9–12 score), *n* (%)	N = 0	N = 30; 6 (20.0%)	n/a
day 20 status–normal (0–1 score), *n* (%)	N = 0	N = 29; 2 (6.9%)	n/a
day 20 status–mild undernutrition (2–4 score), *n* (%)	N = 0	N = 29; 5 (17.2%)	n/a
day 20 status–moderate undernutrition (5–8 score), *n* (%)	N = 0	N = 29; 12 (41.4%)	n/a
day 20 status–severe undernutrition (9–12 score), *n* (%)	N = 0	N = 29; 10 (34.5%)	n/a
Outcomes			
ICU Mortality, *n* (%)	N = 161; 0 (0.0%)	N = 31; 1 (3.2%)	0.161
Use of MV, *n* (%)	N = 106; 70 (66.0%)	N = 31; 20 (64.5%)	0.875
Duration of MV, days	N = 70; Me = 8.0 [2.6; 15.2]	N = 20; Me = 17.0 [7.0; 18.0]	0.013 *
Use of vasopressors/inotropes, *n* (%)	N = 62; 4 (6.5%)	N = 31; 6 (19.4%)	0.067
Nonfatal cardiac arrest, *n* (%)	N = 155; 8 (5.2%)	N = 31; 1 (3.2%)	>0.999
New-onset arrhythmias, *n* (%)	N = 155; 30 (19.4%)	N = 31; 2 (6.5%)	0.082
Acute kidney injury, *n* (%)	N = 155; 36 (23.2%)	N = 31; 2 (6.5%)	0.034 *
Sepsis, *n* (%)	N = 155; 26 (16.8%)	N = 31; 7 (22.6%)	0.440

Abbreviations: †, covariates accounted for propensity score matching; *, differences are statistically significant; Δ, dynamics between two time points (the value of the 20th day minus the value of the first day); ID, insufficient data (less than 3 values in the sample); TBI, traumatic brain injury; BMI, body mass index; WBCs, white blood cells; RBCs, red blood cells; AST, aspartate aminotransferase; ALT, alanine aminotransferase; CRP, C-reactive protein; BUN, blood urea nitrogen; LDL, low-density lipoprotein; HDL, high-density lipoprotein; ICU, intensive care unit; MV, mechanical ventilation.

**Table 2:** Comparison of patients with and without TBI or stroke after PSM.

Parameters	eICU-CRD Patients Without TBI or Stroke (N = 121)	FRCC ICMR Patients With TBI or Stroke (N = 29)	*p*-Value
† Sex (male), *n* (%)	N = 121; 75 (62.0%)	N = 29; 19 (65.5%)	0.724
† Age, years	N = 121; Me = 58.0 [49.0; 64.0]	N = 29; Me = 54.0 [41.0; 68.0]	0.591
† BMI, kg/m2	N = 121; Me = 26.4 [23.2; 30.2]	N = 29; Me = 26.0 [23.5; 29.4]	0.574
Laboratory Tests			
WBCs day 1, 10^9^/L	N = 113; Me = 11.0 [7.7; 16.1]	N = 29; Me = 9.9 [7.0; 12.0]	0.081
WBCs day 20, 10^9^/L	N = 73; Me = 9.3 [6.2; 11.5]	N = 27; Me = 7.7 [6.3; 9.4]	0.265
Δ WBCs day 1–20, 10^9^/L	N = 72; Me = −1.9 [−5.7; 0.9]	N = 27; Me = −1.4 [−2.8; 0.5]	0.326
Δ WBCs day 1–20, %	N = 72; Me = −17.9 [−39.6; 11.2]	N = 27; Me = −16.6 [−29.7; 5.7]	0.383
Hemoglobin day 1, g/L	N = 113; Me = 88.0 [81.0; 102.0]	N = 29; Me = 98.0 [88.0; 106.0]	0.045 *
Hemoglobin day 20, g/L	N = 73; Me = 87.0 [81.0; 97.0]	N = 27; Me = 89.0 [81.0; 102.0]	0.589
Δ Hemoglobin day 1–20, g/L	N = 72; Me = −3.0 [−13.5; 7.5]	N = 27; Me = −5.0 [−18.0; 10.0]	0.414
Δ Hemoglobin day 1–20, %	N = 72; Me = −3.5 [−14.4; 8.2]	N = 27; Me = −6.5 [−17.3; 10.4]	0.423
Hematocrit day 1, %	N = 113; Me = 27.0 [24.9; 31.9]	N = 29; Me = 30.2 [27.6; 32.5]	0.016 *
Hematocrit day 20, %	N = 73; Me = 27.2 [25.1; 30.7]	N = 27; Me = 27.9 [25.5; 31.6]	0.558
Δ Hematocrit day 1–20, %	N = 72; Me = −0.5 [−3.7; 1.8]	N = 27; Me = −2.8 [−5.6; 2.7]	0.270
Δ Hematocrit day 1–20, %	N = 72; Me = −1.7 [−12.5; 6.9]	N = 27; Me = −8.7 [−16.9; 10.9]	0.245
RBCs day 1, 10^12^/L	N = 113; Me = 3.1 [2.7; 3.6]	N = 29; Me = 3.3 [3.0; 3.6]	0.104
RBCs day 20, 10^12^/L	N = 73; Me = 3.0 [2.8; 3.3]	N = 27; Me = 3.2 [2.7; 3.6]	0.233
Δ RBCs day 1–20, 10^12^/L	N = 72; Me = −0.1 [−0.4; 0.3]	N = 27; Me = −0.1 [−0.6; 0.4]	0.826
Δ RBCs day 1–20, %	N = 72; Me = −2.9 [−13.8; 8.9]	N = 27; Me = −3.7 [−15.6; 11.2]	0.863
Lymphocytes day 1, 10^9^/L	N = 74; Me = 1.2 [0.7; 1.7]	N = 29; Me = 1.6 [1.3; 2.0]	0.003 *
Lymphocytes day 20, 10^9^/L	N = 49; Me = 1.4 [0.8; 2.2]	N = 27; Me = 1.5 [1.2; 2.3]	0.551
Δ Lymphocytes day 1–20, 10^9^/L	N = 44; Me = 0.3 [0.0; 0.8]	N = 27; Me = 0.0 [−0.5; 0.1]	0.009 *
Δ Lymphocytes day 1–20, %	N = 44; Me = 30.6 [1.2; 79.3]	N = 27; Me = −1.4 [−27.7; 11.7]	0.008 *
Potassium day 1, mmol/L	N = 113; Me = 3.9 [3.7; 4.2]	N = 29; Me = 3.8 [3.6; 4.0]	0.131
Potassium day 20, mmol/L	N = 73; Me = 4.0 [3.7; 4.3]	N = 27; Me = 3.7 [3.5; 4.2]	0.052
Δ Potassium day 1–20, mmol/L	N = 72; Me = 0.1 [−0.4; 0.6]	N = 27; Me = 0.0 [−0.4; 0.3]	0.489
Δ Potassium day 1–20, %	N = 72; Me = 2.6 [−9.8; 14.3]	N = 27; Me = 0.0 [−10.3; 9.1]	0.418
Sodium day 1, mmol/L	N = 113; Me = 140.0 [136.0; 144.0]	N = 29; Me = 139.1 [135.6; 141.7]	0.663
Sodium day 20, mmol/L	N = 73; Me = 138.0 [135.0; 141.0]	N = 27; Me = 137.6 [134.3; 141.2]	0.724
Δ Sodium day 1–20, mmol/L	N = 72; Me = −1.0 [−6.5; 1.0]	N = 27; Me = −0.1 [−8.7; 5.7]	0.598
Δ Sodium day 1–20, %	N = 72; Me = −0.7 [−4.7; 0.8]	N = 27; Me = −0.1 [−6.4; 4.3]	0.577
Calcium day 1, mmol/L	N = 108; Me = 2.1 [2.0; 2.2]	N = 29; Me = 2.0 [2.0; 2.1]	0.387
Calcium day 20, mmol/L	N = 68; Me = 2.2 [2.0; 2.3]	N = 27; Me = 2.1 [1.9; 2.2]	0.018 *
Δ Calcium day 1–20, mmol/L	N = 66; Me = 0.1 [0.0; 0.2]	N = 27; Me = 0.0 [−0.1; 0.1]	0.030 *
Δ Calcium day 1–20, %	N = 66; Me = 4.6 [−1.3; 10.4]	N = 27; Me = 1.3 [−5.1; 4.4]	0.032 *
Total protein day 1, g/L	N = 55; Me = 56.0 [48.0; 61.0]	N = 29; Me = 60.0 [56.2; 64.0]	0.014 *
Total protein day 20, g/L	N = 25; Me = 57.0 [53.0; 65.0]	N = 27; Me = 56.3 [48.3; 60.1]	0.237
Δ Total protein day 1–20, g/L	N = 21; Me = 6.0 [−4.0; 12.0]	N = 27; Me = −5.6 [−10.8; 0.7]	0.006 *
Δ Total protein day 1–20, %	N = 21; Me = 11.4 [−6.0; 21.4]	N = 27; Me = −7.9 [−18.1; 1.3]	0.005 *
Albumin day 1, g/L	N = 67; Me = 24.0 [20.0; 28.0]	N = 29; Me = 28.3 [24.2; 31.2]	0.011 *
Albumin day 20, g/L	N = 40; Me = 24.0 [20.0; 30.0]	N = 27; Me = 25.9 [22.5; 29.8]	0.252
Δ Albumin day 1–20, g/L	N = 35; Me = 2.0 [−3.0; 5.0]	N = 27; Me = −0.4 [−4.0; 1.5]	0.173
Δ Albumin day 1–20, %	N = 35; Me = 8.3 [−16.7; 22.2]	N = 27; Me = −1.3 [−15.3; 5.3]	0.236
Transferrin day 1, mg/dL	N = 2; 132.0. 181.0	N = 29; Me = 128.0 [92.0; 150.0]	ID
Transferrin day 20, mg/dL	N = 2; 152.0. 124.0	N = 27; Me = 119.0 [79.0; 154.0]	ID
Δ Transferrin day 1–20, mg/dL	N = 1; Me = −57.0	N = 27; Me = −1.0 [−36.0; 12.0]	ID
Δ Transferrin day 1–20, %	N = 1; Me = −31.5	N = 27; Me = −0.8 [−38.2; 20.9]	ID
AST day 1, U/L	N = 58; Me = 36.5 [18.0; 78.0]	N = 29; Me = 29.8 [21.6; 40.2]	0.380
AST day 20, U/L	N = 27; Me = 31.0 [21.0; 64.0]	N = 27; Me = 23.6 [20.2; 52.6]	0.333
Δ AST day 1–20, U/L	N = 25; Me = −6.0 [−17.0; 7.0]	N = 27; Me = −0.5 [−14.3; 7.0]	0.721
Δ AST day 1–20, %	N = 25; Me = −17.1 [−31.9; 45.5]	N = 27; Me = −1.8 [−35.2; 74.5]	0.920
ALT day 1, U/L	N = 57; Me = 34.0 [22.0; 80.0]	N = 29; Me = 27.6 [21.8; 39.2]	0.267
ALT day 20, U/L	N = 27; Me = 34.0 [17.0; 65.0]	N = 27; Me = 25.1 [20.7; 39.8]	0.473
Δ ALT day 1–20, U/L	N = 24; Me = −0.5 [−67.5; 10.0]	N = 27; Me = −0.6 [−6.5; 4.7]	0.406
Δ ALT day 1–20, %	N = 24; Me = −2.8 [−58.2; 47.1]	N = 27; Me = −2.8 [−30.5; 41.4]	0.546
Total bilirubin day 1, μmol/L	N = 58; Me = 13.7 [6.8; 42.8]	N = 29; Me = 11.9 [9.6; 16.2]	0.260
Total bilirubin day 20, μmol/L	N = 27; Me = 8.6 [6.8; 42.8]	N = 27; Me = 11.3 [8.1; 13.5]	0.574
Δ Total bilirubin day 1–20, μmol/L	N = 25; Me = −5.1 [−22.2; 1.7]	N = 27; Me = −1.6 [−4.2; 2.5]	0.245
Δ Total bilirubin day 1–20, %	N = 25; Me = −33.3 [−72.7; 1.5]	N = 27; Me = −15.6 [−31.3; 22.6]	0.184
Direct bilirubin day 1, μmol/L	N = 29; Me = 3.4 [0.0; 15.4]	N = 28; Me = 2.7 [1.5; 3.7]	0.169
Direct bilirubin day 20, μmol/L	N = 14; Me = 1.7 [0.0; 8.6]	N = 27; Me = 2.4 [1.3; 3.2]	0.776
Δ Direct bilirubin day 1–20, μmol/L	N = 9; Me = −1.7 [−20.5; 0.0]	N = 26; Me = −0.1 [−1.8; 0.7]	0.051
Δ Direct bilirubin day 1–20, %	N = 6; Me = −85.4 [−100.0; −50.0]	N = 26; Me = −1.9 [−55.6; 40.7]	0.001 *
CRP day 1, mg/L	N = 1; 151.0	N = 29; Me = 77.3 [33.5; 126.9]	ID
CRP day 20, mg/L	N = 2; 87.6	N = 27; Me = 43.1 [21.1; 123.6]	ID
Δ CRP day 1–20, mg/L	N = 0	N = 27; Me = −22.3 [−69.3; 18.6]	n/a
Δ CRP day 1–20, %	N = 0	N = 27; Me = −49.6 [−75.0; 77.3]	n/a
BUN day 1, mmol/L	N = 113; Me = 10.7 [5.7; 18.9]	N = 29; Me = 5.8 [3.6; 10.4]	0.001 *
BUN day 20, mmol/L	N = 73; Me = 10.4 [5.0; 15.4]	N = 27; Me = 4.6 [2.4; 7.9]	<0.001 *
Δ BUN day 1–20, mmol/L	N = 72; Me = −0.4 [−8.2; 3.0]	N = 27; Me = −0.9 [−6.1; 1.4]	0.969
Δ BUN day 1–20, %	N = 72; Me = −4.3 [−49.6; 40.4]	N = 27; Me = −22.7 [−63.5; 102.4]	0.629
Creatinine day 1, μmol/L	N = 111; Me = 99.9 [53.0; 229.8]	N = 29; Me = 59.1 [50.9; 90.0]	0.008 *
Creatinine day 20, μmol/L	N = 71; Me = 88.4 [55.7; 185.6]	N = 27; Me = 59.3 [47.9; 92.0]	0.041 *
Δ Creatinine day 1–20, μmol/L	N = 69; Me = −13.3 [−67.2; 9.7]	N = 27; Me = −2.9 [−14.3; 14.2]	0.085
Δ Creatinine day 1–20, %	N = 69; Me = −12.3 [−41.5; 17.2]	N = 27; Me = −4.7 [−18.8; 38.0]	0.106
Glucose day 1, mmol/L	N = 113; Me = 7.2 [5.6; 8.7]	N = 28; Me = 5.2 [4.5; 5.7]	<0.001 *
Glucose day 20, mmol/L	N = 73; Me = 5.8 [5.0; 7.2]	N = 27; Me = 4.8 [4.5; 5.4]	0.001 *
Δ Glucose day 1–20, mmol/L	N = 72; Me = −0.8 [−2.2; −0.1]	N = 26; Me = −0.2 [−1.2; 0.6]	0.024 *
Δ Glucose day 1–20, %	N = 72; Me = −12.2 [−26.7; −2.2]	N = 26; Me = −4.0 [−19.5; 17.1]	0.039 *
Total cholesterol day 1, mmol/L	N = 1; 3.4	N = 28; Me = 3.9 [3.2; 4.5]	ID
Total cholesterol day 20, mmol/L	N = 1; 3.2	N = 27; Me = 3.5 [2.8; 4.8]	ID
Δ Total cholesterol day 1–20, mmol/L	N = 0	N = 26; Me = −0.2 [−1.1; 0.7]	n/a
Δ Total cholesterol day 1–20, %	N = 0	N = 26; Me = −7.4 [−22.3; 20.8]	n/a
LDL day 1, mmol/L	N = 1; 1.8	N = 29; Me = 2.6 [2.2; 3.2]	ID
LDL day 20, mmol/L	N = 1; 1.7	N = 27; Me = 2.4 [1.7; 3.2]	ID
Δ LDL day 1–20, mmol/L	N = 0	N = 27; Me = −0.5 [−0.9; 0.4]	n/a
Δ LDL day 1–20, %	N = 0	N = 27; Me = −17.3 [−35.2; 13.2]	n/a
HDL day 1, mmol/L	N = 1; 1.2	N = 29; Me = 0.7 [0.6; 0.8]	ID
HDL day 20, mmol/L	N = 1; 0.6	N = 27; Me = 0.8 [0.5; 1.0]	ID
Δ HDL day 1–20, mmol/L	N = 0	N = 27; Me = 0.1 [−0.1; 0.2]	n/a
Δ HDL day 1–20, %	N = 0	N = 27; Me = 6.9 [−14.3; 36.6]	n/a
Cortisol day 1, nmol/L	N = 3; Me = 309.0 [256.6; 612.5]	N = 29; Me = 402.3 [319.4; 489.9]	0.714
Cortisol day 20, nmol/L	N = 0	N = 27; Me = 399.9 [249.4; 542.1]	n/a
Δ Cortisol day 1–20, nmol/L	N = 0	N = 27; Me = −38.8 [−153.9; 115.3]	n/a
Δ Cortisol day 1–20, %	N = 0	N = 27; Me = −13.2 [−33.3; 62.0]	n/a
Prognostic Nutritional Index (PNI)			
day 1	N = 51; Me = 30.8 [26.5; 36.9]	N = 29; Me = 37.6 [30.6; 41.5]	0.006 *
day 20	N = 32; Me = 31.7 [27.5; 38.3]	N = 27; Me = 36.0 [28.0; 40.3]	0.338
Δ day 1–20	N = 25; Me = 3.5 [−0.9; 7.7]	N = 27; Me = −1.3 [−4.1; 3.8]	0.047 *
day 1 status–normal (≥45), *n* (%)	N = 51; 2 (3.9%)	N = 29; 4 (13.8%)	0.182
day 1 status–mild malnutrition (40–44.9), *n* (%)	N = 51; 3 (5.9%)	N = 29; 6 (20.7%)	0.065
day 1 status–severe malnutrition (<40), *n* (%)	N = 51; 46 (90.2%)	N = 29; 19 (65.5%)	0.007*
day 20 status–normal (≥45), *n* (%)	N = 32; 3 (9.4%)	N = 27; 1 (3.7%)	0.617
day 20 status–mild malnutrition (40–44.9), *n* (%)	N = 32; 3 (9.4%)	N = 27; 6 (22.2%)	0.277
day 20 status–severe malnutrition (<40), *n* (%)	N = 32; 26 (81.3%)	N = 27; 20 (74.1%)	0.508
Controlling Nutritional Status (COUNT)			
day 1, score	N = 0	N = 28; Me = 6.5 [4.0; 8.0]	n/a
day 20, score	N = 0	N = 27; Me = 7.0 [5.0; 9.0]	n/a
Δ day 1–20, score	N = 0	N = 26; Me = 0.5 [−1.0; 2.0]	n/a
day 1 status–normal (0–1 score), *n* (%)	N = 0	N = 28; 2 (7.1%)	n/a
day 1 status–mild undernutrition (2–4 score), *n* (%)	N = 0	N = 28; 7 (25.0%)	n/a
day 1 status–moderate undernutrition (5–8 score), *n* (%)	N = 0	N = 28; 13 (46.4%)	n/a
day 1 status–severe undernutrition (9–12 score), *n* (%)	N = 0	N = 28; 6 (21.4%)	n/a
day 20 status–normal (0–1 score), *n* (%)	N = 0	N = 27; 2 (7.4%)	n/a
day 20 status–mild undernutrition (2–4 score), *n* (%)	N = 0	N = 27; 4 (14.8%)	n/a
day 20 status–moderate undernutrition (5–8 score), *n* (%)	N = 0	N = 27; 11 (40.7%)	n/a
day 20 status–severe undernutrition (9–12 score), *n* (%)	N = 0	N = 27; 10 (37.0%)	n/a
Outcomes			
ICU Mortality, *n* (%)	N = 121; 0 (0.0%)	N = 29; 1 (3.4%)	0.193
Use of MV, *n* (%)	N = 84; 54 (64.3%)	N = 29; 20 (69.0%)	0.648
Duration of MV, days	N = 54; Me = 8.0 [2.7; 16.2]	N = 20; Me = 17.0 [7.0; 18.0]	0.030 *
Use of vasopressors/inotropes, *n* (%)	N = 47; 4 (8.5%)	N = 29; 6 (20.7%)	0.167
Nonfatal cardiac arrest, *n* (%)	N = 115; 7 (6.1%)	N = 29; 1 (3.4%)	>0.999
New-onset arrhythmias, *n* (%)	N = 115; 22 (19.1%)	N = 29; 2 (6.9%)	0.164
Acute kidney injury, *n* (%)	N = 115; 31 (27.0%)	N = 29; 2 (6.9%)	0.022*
Sepsis, *n* (%)	N = 115; 20 (17.4%)	N = 29; 6 (20.7%)	0.680

Abbreviations: †, covariates accounted for propensity score matching; *, differences are statistically significant; Δ, dynamics between two time points (the value of the 20th day minus the value of the first day); ID, insufficient data (less than 3 values in the sample); TBI, traumatic brain injury; BMI, body mass index; WBCs, white blood cells; RBCs, red blood cells; AST, aspartate aminotransferase; ALT, alanine aminotransferase; CRP, C-reactive protein; BUN, blood urea nitrogen; LDL, low-density lipoprotein; HDL, high-density lipoprotein; ICU, intensive care unit; MV, mechanical ventilation.

## Data Availability

Restrictions apply to the availability of these data.

## Supplementary Materials

The following supporting information can be downloaded at: https://www.mdpi.com/article/10.3390/nu17142302/s1, Table S1: STROBE statement—checklist; Table S2: The analysis of the effect of the duration of mechanical ventilation on the dynamics of total protein—simple linear regression; Figure S1: The analysis of the effect of the duration of mechanical ventilation on the dynamics of total protein—scatter plot (A—for Δ total protein day 1–20, g/L; B—for Δ total protein day 1–20, %).

## Abbreviations

The following abbreviations are used in this manuscript:

%bias	Standardized percentage bias across covariates
ALT	Alanine aminotransferase
AST	Aspartate aminotransferase
BMI	Body mass index
BUN	Blood urea nitrogen
COUNT	Controlling Nutritional Status
CRP	C-reactive protein
eICU-CRD	eICU Collaborative Research Database
FRCC ICMR	Federal Research and Clinical Center of Intensive Care Medicine and Rehabilitology
HDL	High-density lipoprotein
ICU	Intensive care unit
ID	insufficient data
LDL	Low-density lipoprotein
PEEP	Positive end-expiratory pressure
PNI	Prognostic Nutritional Index
PSM	Propensity score matching
RBCs	Red blood cells
TBI	Traumatic brain injury
WBCs	White blood cells
