# Supplementary material for: The Neurological Metabolic Phenotype in Prolonged/Chronic Critical Illness: Propensity Score Matched Analysis of Nutrition and Outcomes
Source: Nutrients. 2025 Jul 12;17(14):2302. doi: 10.3390/nu17142302 (PMC12298954; doi:10.3390/nu17142302)

# Supplementary Materials

## The neurological metabolic phenotype in prolonged/chronic critical illness: propensity score matched analysis of nutrition and outcomes

**Levan B. Berikashvili[1], Alexander E. Shestopalov[1], Petr A. Polyakov[1], Alexandra V. Yakovleva[1], Mikhail Ya. Yadgarov[1,2], Ivan V. Kuznetsov[1], Mohammad Tarek S.M. Said[1], Ivan V. Sergeev[1], Alexey A. Yakovlev[1], Valery V. Likhvantsev[1]**

1 Federal Research and Clinical Center of Intensive Care Medicine and Rehabilitology, Moscow 107031, Russia;
2 V. M. Gorbatov Federal Research Center for Food Systems, Russian Academy of Sciences, Moscow 109316, Russia.
* Correspondence: lberikashvili@fnkcrr.ru

**Table S1.** STROBE Statement—Checklist of items that should be included in reports of cohort studies

| | Item No | Recommendation | Page No |
|---|---|---|---|
| **Title and abstract** | 1 | (*a*) Indicate the study's design with a commonly used term in the title or the abstract | 2 |
| | | (*b*) Provide in the abstract an informative and balanced summary of what was done and what was found | 1 |
| **Introduction** | | | |
| Background/rationale | 2 | Explain the scientific background and rationale for the investigation being reported | 2 |
| Objectives | 3 | State specific objectives, including any prespecified hypotheses | 2 |
| **Methods** | | | |
| Study design | 4 | Present key elements of study design early in the paper | 1, 2 |
| Setting | 5 | Describe the setting, locations, and relevant dates, including periods of recruitment, exposure, follow-up, and data collection | 2, 3 |
| Participants | 6 | (*a*) Give the eligibility criteria, and the sources and methods of selection of participants. Describe methods of follow-up | 3 |
| | | (*b*) For matched studies, give matching criteria and number of exposed and unexposed | 3, 4 |
| Variables | 7 | Clearly define all outcomes, exposures, predictors, potential confounders, and effect modifiers. Give diagnostic criteria, if applicable | 3 |
| Data sources/ measurement | 8* | For each variable of interest, give sources of data and details of methods of assessment (measurement). Describe comparability of assessment methods if there is more than one group | 3 |
| Bias | 9 | Describe any efforts to address potential sources of bias | 3, 4 |
| Study size | 10 | Explain how the study size was arrived at | 2 (As this was a pilot |

| | | | |
|---|---|---|---|
| | | | observational study, no formal sample size or power calculations were performed) |
| Quantitative variables | 11 | Explain how quantitative variables were handled in the analyses. If applicable, describe which groupings were chosen and why | 3 |
| Statistical methods | 12 | (*a*) Describe all statistical methods, including those used to control for confounding | 3, 4 |
| | | (*b*) Describe any methods used to examine subgroups and interactions | Not performed (without examine subgroups) |
| | | (*c*) Explain how missing data were addressed | Not performed (available-data analysis) |
| | | (*d*) If applicable, explain how loss to follow-up was addressed | Not performed (short follow-up in ICU) |
| | | (*e*) Describe any sensitivity analyses | Not performed |
| **Results** | | | |
| Participants | 13* | (a) Report numbers of individuals at each stage of study—eg numbers potentially eligible, examined for eligibility, confirmed eligible, included in the study, completing follow-up, and analysed | 4, Figure 1 |
| | | (b) Give reasons for non-participation at each stage | 3 |
| | | (c) Consider use of a flow diagram | Figure 1 |
| Descriptive data | 14* | (a) Give characteristics of study participants (eg demographic, clinical, social) and information on exposures and potential confounders | 5 |
| | | (b) Indicate number of participants with missing data for each variable of interest | 5 |
| | | (c) Summarise follow-up time (eg, average and total amount) | 3 (fixed 20-day observation) |
| Outcome data | 15* | Report numbers of outcome events or summary measures over time | 9, 10 |
| Main results | 16 | (*a*) Give unadjusted estimates and, if applicable, confounder-adjusted estimates and their precision (eg, 95% confidence interval). Make clear which confounders were adjusted for and why they were included | 6, 9 |

| | | | |
|---|---|---|---|
| | | (*b*) Report category boundaries when continuous variables were categorized | Not performed |
| | | (*c*) If relevant, consider translating estimates of relative risk into absolute risk for a meaningful time period | Not performed |
| Other analyses | 17 | Report other analyses done—eg analyses of subgroups and interactions, and sensitivity analyses | Not performed |
| **Discussion** | | | |
| Key results | 18 | Summarise key results with reference to study objectives | 10, 11 |
| Limitations | 19 | Discuss limitations of the study, taking into account sources of potential bias or imprecision. Discuss both direction and magnitude of any potential bias | 12 |
| Interpretation | 20 | Give a cautious overall interpretation of results considering objectives, limitations, multiplicity of analyses, results from similar studies, and other relevant evidence | 12 |
| Generalisability | 21 | Discuss the generalisability (external validity) of the study results | 12 |
| **Other information** | | | |
| Funding | 22 | Give the source of funding and the role of the funders for the present study and, if applicable, for the original study on which the present article is based | 12 |

*Give information separately for exposed and unexposed groups.

**Note:** An Explanation and Elaboration article discusses each checklist item and gives methodological background and published examples of transparent reporting. The STROBE checklist is best used in conjunction with this article (freely available on the Web sites of PLoS Medicine at http://www.plosmedicine.org/, Annals of Internal Medicine at http://www.annals.org/, and Epidemiology at http://www.epidem.com/). Information on the STROBE Initiative is available at http://www.strobe-statement.org.

**Table S2.** The analysis of the effect of the duration of mechanical ventilation on the dynamics of total protein - simple linear regression.

| Model | Non-standardized coefficients | | 95.0% Confidence interval for B | | Standardized coefficients | Significance |
|---|---|---|---|---|---|---|
| | B | The standard error | The lower limit | The upper limit | Beta | |
| ***Dependent variable: Δ Total protein day 1-20, g/L*** | | | | | | |
| Constant | -4.079 | 5.356 | -15.033 | 6.875 | 0 | 0.452 |
| Duration of MV, days | 0.052 | 0.343 | -0.650 | 0.754 | 0.028 | 0.880 |
| ***Dependent variable: Δ Total protein day 1-20, %*** | | | | | | |
| Constant | -6.435 | 9.032 | -24.909 | 12.038 | 0 | 0.482 |
| Duration of MV, days | 0.147 | 0.579 | -1.037 | 1.330 | 0.047 | 0.802 |

**Figure S1.** The analysis of the effect of the duration of mechanical ventilation on the dynamics of total protein - Scatter plot (A - for Δ Total protein day 1-20, g/L; B - for Δ Total protein day 1-20, %).

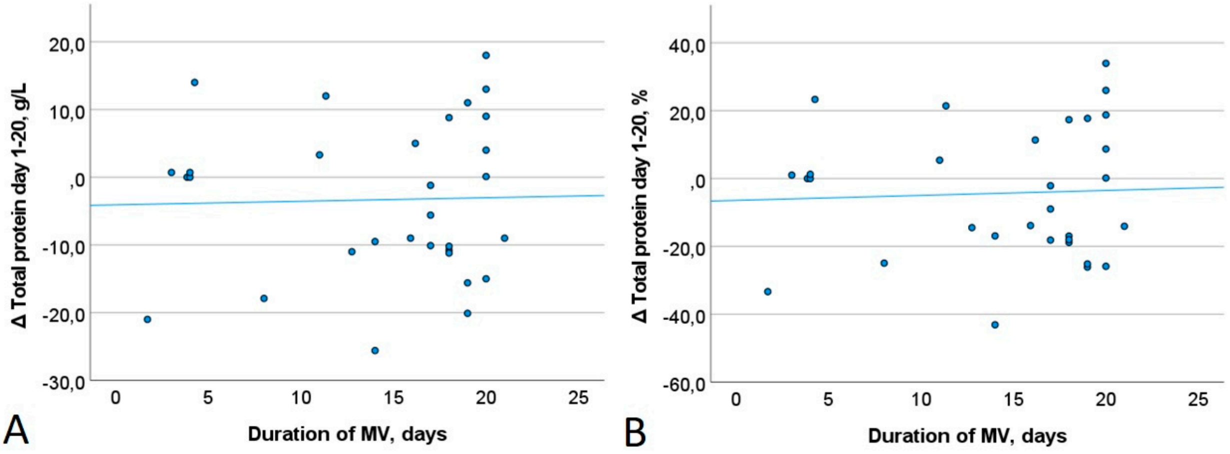

Supplement: Supplementary file 1 [file nutrients-17-02302-s001.zip › nutrients-3737726-supplementary.pdf]
